# Mechanistic insights into the cytotoxic and apoptotic effects of 1-butyl-3-methylimidazolium bromide and 1-butylpyridinium bromide on human cell lines

**DOI:** 10.2478/aiht-2025-76-3966

**Published:** 2025-09-30

**Authors:** Lu Gao, Charles Obinwanne Okoye, Bonaventure Chidi Ezenwanne, Jianxiong Jiang, Guizhu Wu, Lin Yu, Daolin Du, Yonglai Xue

**Affiliations:** Jiangsu University School of Environment and Safety Engineering, Biofuels Institute, Zhenjiang, China; Jiangsu University School of Life Sciences, Zhenjiang, China; University of Nigeria Faculty of Biological Sciences, Department of Zoology and Environmental Biology, Nsukka, Nigeria; Jiangsu University School of Environment and Safety Engineering, Institute of Environment and Ecology, Zhenjiang, China; Tianjin Medical University School of Basic Medical Sciences, Department of Biochemistry and Molecular Biology, Tianjin, China; Tianjin Medical University Key Laboratory of the Educational Ministry of China, Tianjin, China; Jiangsu University Jingjiang College, Zhenjiang, China

**Keywords:** anti-proliferative effects, apoptosis, cell cycle arrest, cytotoxicity, HeLa cells, HEK293T cells, ionic liquids, MCF-7 cells, antiproliferativno djelovanje, apoptoza, citotoksičnost, HEK293T stanice, HeLa stanice, ionske tekućine, MCF-7 stanice, zaustavljanje staničnog ciklusa

## Abstract

Ionic liquids (ILs) are a novel class of salts with growing industrial applications due to their tunable physicochemical properties. However, their popularity has given rise to concerns about their cytotoxic potential. This study investigates the cytotoxic, apoptotic, and cell cycle effects of two ILs, namely 1-butyl-3-methylimidazolium bromide ([Bmim]Br) and 1-butylpyridinium bromide ([Bpy]Br), on three human cell lines: HeLa (cervical cancer), MCF-7 (breast cancer), and HEK293T (human embryonic kidney). Using real-time cell analysis (RTCA), we determined that the half-maximal inhibition concentrations (IC_50_) for [Bmim]Br were 841.86 μmol/L in MCF-7, 538.38 μmol/L in HeLa, and 654.78 μmol/L in HEK293T cells. Respective [Bpy]Br IC_50_ values were 341.74 μmol/L, 333.27 μmol/L, and 328.98 μmol/L. Flow cytometry revealed that both ILs induced dose-dependent apoptosis and that [Bpy]Br showed stronger pro-apoptotic effects. At 1000 μmol/L, [Bpy]Br reduced live cell population to 33.86 % in MCF-7 and to 38.32 % in HeLa cells. Both ILs induced the G0/G1 phase arrest and significantly suppressed the expression of cyclin D1, CDK2, and CDK4 at both mRNA and protein levels. MTT and Transwell assays further confirmed inhibited cell proliferation and migration, particularly in MCF-7 and HeLa cells. These findings demonstrate that [Bmim]Br and [Bpy]Br inhibit cell growth by triggering apoptosis and by interfering with cell cycle progression. Stronger effects observed with [Bpy]Br suggest its therapeutic potential, but given the toxicity of both ILs in non-cancerous HEK293T cells, further research is necessary to evaluate their biosafety and long-term effects.

## Abstract (hr)

Ionske tekućine nova su klasa soli čija se rastuća primjena u industriji može zahvaliti mogućnosti prilagodbe njihovih fizikalno-kemijskih svojstava. No, sa sve većom popularnosti raste i zabrinutost zbog njihove moguće citotoksičnosti. Cilj je ovog istraživanja bio utvrditi citotoksično i apoptotsko djelovanje dviju takvih ionskih tekućina – 1-butil-3-metilimidazol bromida ([Bmim]Br) i 1-butilpiridin bromida ([Bpy]Br) – na ljudske stanične linije HeLa (rak vrata maternice), MCF-7 (rak dojki) i HEK293T (ljudske embrijske bubrežne stanice) te na njihov stanični ciklus. Analizom stanica u stvarnom vremenu (engl. *real-time cell analysis*, krat. *RTCA*) utvrđeno je da su koncentracije [Bmim]Br, potrebne za 50-postotnu inhibiciju (IC_50_) MCF-7 stanica, bile 841,86 μmol/L, HeLa stanica 538,38 μmol/L te HEK293T stanica 654,78 μmol/L. Odgovarajuće IC_50_ vrijednosti za [Bpy]Br bile su 341,74 μmol/L, 333,27 μmol/L i 328,98 μmol/L. Protočna citometrija pokazala je da obje ionske tekućine izazivaju apoptozu ovisnu o dozi te da [Bpy]Br ima izraženije apoptotsko djelovanje. Pri koncentraciji od 1000 μmol/L [Bpy]Br je smanjio udio živih MCF-7 stanica na 33,86 %, odnosno HeLa stanica na 38,32 %. Obje ionske tekućine zaustavile su stanični ciklus u G0/G1 fazi te značajno smanjile ekspresiju ciklina D1, CDK2 i CDK4 na razini mRNA i proteina. MTT i test proveden primjenom tzv. „transwell“ propusnih nosača dodatno su potvrdili smanjenu proliferaciju i migraciju stanica, osobito u MCF-7 i HeLa linijama. Ovi rezultati pokazuju da [Bmim]Br i [Bpy]Br inhibiraju rast stanica raka izazivanjem apoptoze i ometanjem staničnog ciklusa. Snažnije djelovanje [Bpy]Br upućuje na njegov terapijski potencijal, no zbog toksičnosti za nekancerogene HEK239T stanice potrebna su daljnja istraživanja radi procjene sigurnosti njegove primjene i dugoročnih učinaka.

Ionic liquids (ILs) are a novel class of low-temperature molten salts composed of discrete cations and anions, known for their unique and versatile properties (1,2,3) such as non-flammability, low vapour pressure, non-volatility, good electrical conductivity, high thermal stability, and wide electrochemical stability (4). Their chemical, physical, and biological properties can be adjusted or “tuned” by changing cations and anions, functionalising alkyl groups, and altering the length of the alkyl chains in their composition (5, 6). ILs have elicited much interest as alternatives to organic solvents in various industrial chemical processes, including synthesis and separation/extraction techniques. In addition, they have been explored in biological applications such as enzyme catalysis, drug delivery, antimicrobial formulations, and biomolecule stabilisation (7).

There have been reports of the eco-friendly nature of ILs due to their high boiling point and low volatility (8). However, studies on the toxic effects of various ILs have demonstrated that they can still affect the environment (9), water in particular, due to their high water solubility and low biodegradability (10). In aquatic environments, they are toxic to a number of organisms, such as bacteria, crustaceans, and fish (11). Over the past two decades, extensive research has been focused on the toxicity of ILs in living cells (12) to evaluate the risks linked to their potential industrial applications and to create strategies for designing more environmentally friendly ILs.

Among the various ILs studied, 1-butyl-3-methylimidazolium bromide ([Bmim]Br) and 1-butylpyridinium bromide ([Bpy]Br) have attracted attention due to their structural stability, solubility in aqueous media, and tunable biological activities (13, 14). [Bmim]Br, an imidazolium-based IL, is often used as a solvent and catalyst in chemical synthesis (15), while [Bpy]Br, a pyridinium-based IL, has applications in electrochemical and pharmaceutical research (16). Their cationic head groups interact with cellular membranes and can disrupt membrane integrity, mitochondrial function, and signalling pathways (17). Despite their utility, both ILs have demonstrated cytotoxic and genotoxic properties in various biological models (18) and human cell lines (19,20,21,22,23). Recent studies indicate that these ILs engage with cells through various mechanisms. These include mitochondrial permeabilisation and dysfunction, production of reactive oxygen species, changes in lipid distribution and the viscoelastic properties of cell membranes, disruption of cell and nuclear membranes, functional modifications of transmembrane and cytoplasmic proteins, damage to chloroplasts in plants, fragmentation of DNA, and alterations in signalling pathways (12, 24, 25).

However, little is still known about how their cytotoxicity affects the cell cycle in human cells. Cell cycle has four stages: Gap 1 (G1), DNA synthesis (S), Gap 2 (G2), and finally, cell division (M). This cycle is strictly regulated by a set of proteins known as cyclins, along with their partner cyclin-dependent kinases (CDKs) (26). The aim of our study was therefore to take a closer look at how [Bmim][Br] and [Bpy][Br] affect the cell cycle and induce cytotoxicity and apoptosis in human breast cancer cells (MCF-7), human cervical cancer cells (HeLa), and healthy human embryonic kidney cells (HEK293T), as well as to gain an insight into the mechanisms of IL-induced cytotoxicity.

## MATERIALS AND METHODS

[Bmim]Br (CAS No. 85100-77-2) and [Bpy]Br (CAS No. 874-80-6) were purchased/obtained from Aladdin (Shanghai, China) (Table 1).

**Table 1:** Names and general structures of the tested ionic liquids

**Name**	**Abbreviation**	**Structure**
1-butyl-3-methylimidazolium bromide	[Bmim][Br]	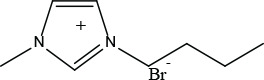
1-butyl-3-methylpyridinium bromide	[Bpy][Br]	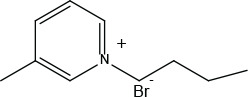

MCF-7, HeLa, and HEK293T cell lines were purchased from the Cell Bank of Shanghai Institute of Biochemistry and Cell Biology (SIBCB), with serial numbers SCSP-531 (CSTR:19375.09.3101HUMSCSP531), TCHu187 (CSTR:19375.09. 3101HUMTCHu187), and SCSP-502 (CSTR:19375.09. 3101HUMSCSP502), respectively. The cell lines were cultured in Dulbecco’s Modified Eagle Medium (DMEM) (Thermo Fisher Scientific Inc., Waltham, MA, USA) supplemented with 10 % foetal bovine serum (Gibco, Shanghai, China) at 37 °C, 5 % CO_2_, and 100 % humidity for routine passaging.

### IC_50_ determination

The cell lines were treated with [Bmim]Br and [Bpy]Br in the concentration range of 10–5000 μmol/L for 6 h. Their half-maximal inhibition concentration (IC_50_) was measured with real-time cell analysis (RTCA) iCELLigence system (ACEA Bioscience, San Diego, CA, USA) as per manufacturer’s instructions. The cells were trypsinised and resuspended at a concentration of 1×10^5^ cells/mL. A 300 μL aliquot of the cell suspension was transferred to each well of an E-Plate L8 (ACEA Bioscience). The extent of cell receptor activity was monitored every half hour for 24 h as described in detail elsewhere (27).

### Cell proliferation and long-term cytotoxicity measurements

MCF-7, HeLa, and HEK293T cell lines were seeded equally in a 96-well plate and treated with [Bmim] Br and [Bpy] Br concentrations ranging from 1 μmol/L to 5000 μmol/L for 0, 1, 2, 3, and 4 days to determine cell proliferation and long-term toxicity. The supernatants were discarded, and 200 μL of culture medium containing 0.5 % 3-(4,5-dimethylthiazol-2-yl)-2,5-diphenyltetrazoliumbromide (MTT) (Sigma, St. Louis, MO, USA) added to each well. After culturing for another 2 h, the culture medium was carefully removed and 150 μL of dimethyl sulphoxide (DMSO; Sigma) added to each well. The cells were placed in an incubator and vortexed at a low speed for 10 min to fully dissolve the crystals. The absorbance of each well was measured at 490 nm using a Varioskan Flash Multimode Reader (Thermo Fisher).

### Measurement of cell motility

To determine the effects of the two ILs on cell motility we used the Transwell assay. MCF-7, HeLa, and HEK293T cells were treated with [Bmim]Br and [Bpy]Br at the concentrations of 100, 600, and 3000 μmol/L each for four days. The cells were seeded in Transwell inserts with 8 μm pore size (FACSCalibur flow cytometer, BD Biosciences, Franklin Lakes, NJ, USA) at 5×10^5^ cells per well. After 24 h, the cells that migrated through the filter were fixed and stained with 10 % crystal violet (Sangon Biotech, Shanghai, China). Cell motility is represented in the plot as the mean number of migrated cells from five random fields for each chamber.

### Assessment of apoptosis and cell cycle progression

To assess apoptosis, HeLa and MCF-7 cells were treated with different concentrations of [Bmim]Br (0, 250, 750, and 1500 μmol/L) and [Bpy]Br (100, 500, and 1000 μmol/L) for 6 h, based on the IC_50_ values and dose-dependent effects presented in results (Table 3). After treatment, apoptosis was assessed using Annexin V-FITC/PI double staining (Beyotime Biotechnology, China) in line with the manufacturer’s instructions and described in detail elsewhere (28). Briefly, 1×10^5^ cells were harvested, washed twice with cold phosphate-buffered saline, and resuspended in 500 μL of binding buffer containing 5 μL of Annexin V-FITC and 5 μL of propidium iodide. After 15 min of incubation in the dark, cells were analysed with flow cytometry (BD Accuri C6 Plus, BD Biosciences) to determine the percentages of live, early apoptotic, late apoptotic, and dead cells.

For cell cycle analysis, cells were fixed with 70 % ethanol at 4 °C for at least 8 h, treated with 10 mg/mL RNase solution (Sigma) at 37 °C for 30 min, stained with 40 μg/mL propidium iodide for another 30 min, and analysed with a Guava easyCyte flow cytometer (Merck Millipore, Burlington, MA, USA).

### Determination of protein levels

Apoptosis-related and cell cycle proteins were determined with the Western blot. Cellular protein dissolved with the radioimmunoprecipitation assay (RIPA) buffer with phenylmethylsulfonyl fluoride (PMSF) (Sigma) was used, and protein concentrations measured with the bicinchoninic acid assay (BCA). Total proteins (35 μg per sample) were extracted from HeLa, MCF-7, and HEK293T cells treated with 1500 μmol/L [Bmim]Br or 1000 μmol/L [Bpy]Br, separated by electrophoresis on a 12 % sodium dodecyl sulphate polyacrylamide gel and transferred to membranes. The membranes were probed with primary antibodies against cyclin D1, cyclin E1, CDK2, and CDK4 (1:1000 dilution) (CST, USA) as described elsewhere (25). β-actin was used as a loading control (1:20000 dilution; Abcam, Cambridge, UK; and 1:6000 dilution; Sigma).

### RNA extraction and quantitative RT-PCR

Total RNA was isolated from cells using the guanidinium thiocyanate-phenol-chloroform extraction (TRIzol, Thermo Fisher). Primers were synthesised by Sangon Biotechnology Co. Ltd. (Shanghai, China). Complementary DNA (cDNA) was generated using a RevertAid cDNA Synthesis Kit (Thermo Fisher). For the quantitative reverse transcription polymerase chain reactions (qRTPCR) we used the ^ΔΔ^Ct method run with a FastStart Universal SYBR Green Master mix (Roche, Nutley, NJ, USA) on a StepOne™ Real-Time PCR System (Applied Biosystems, USA). The qRT-PCR conditions were 95 °C for 2 min, followed by 40 cycles at 95 °C for 30 s, and finally one cycle at 60 °C for 1 min. Glyceraldehyde 3-phosphate dehydrogenase (GAPDH) was used as the housekeeping gene, and mRNA data are presented as the ratio between the target and GAPDH.

### Statistical analysis

Numerical data are presented as means ± standard deviations (SD) of at least three independent experiments. Student’s *t*-test was used for pairwise comparisons of each treatment group with the untreated control. One-way ANOVA was used for comparisons between [Bmim]Br- and [Bpy]Br-treated groups at corresponding concentrations, as well as for multiple-group comparisons across concentrations. Statistical significance was set at p<0.05. All analyses were run on the SPSS 16.0 software (IBM, Armonk, NY, USA).

## RESULTS

[Bmim]Br and [Bpy]Br acute cytotoxicity in human cell lines Table 2 shows the IC_50_ values of [Bmim]Br and [Bpy]Br in HeLa, MCF-7, and HEK293T cells. The effects of the two ILs were concentration-dependent, and [Bpy]Br was significantly more cytotoxic than [Bmim]Br.

**Table 2:** Comparison of [Bmim]Br and [Bpy]Br half-maximal inhibition (IC_50_) in different human cell lines

**Cell type**	**IC_50_ (μmol/L)**		
	[Bmim]Br	[Bpy]Br	
HeLa	841.86±21.03	341.74±13.61*	
MCF-7	538.38±10.54	333.27±19.18*	
HEK293-T	654.78±22.6	328.98±26.49*	

* significant difference from [Bmim]Br in the same cell type (p<0.05; ANOVA)

### Long-term cytotoxicity of [Bmim]Br and [Bpy]Br and effects on cell proliferation

**Figure 1:**
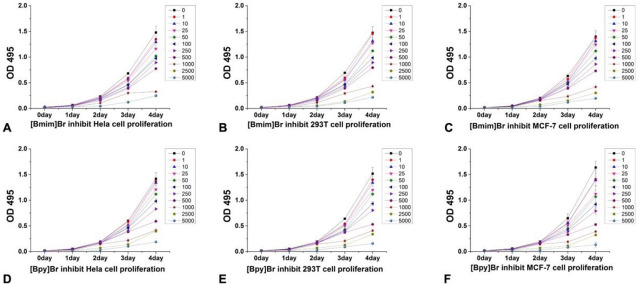
Proliferation curves for [Bmim]Br in A) MCF-7; B) HeLa; and C) HEK293T cells and for [Bpy]Br in D) HeLa; E) HEK293T; and F) MCF-7 cells

Figure 1 shows the inhibitory effects of [Bmim]Br and [Bpy]Br on cell proliferation in HeLa, HEK293T, and MCF-7 cell lines over four days. Both compounds exhibited concentration- and time-dependent cytotoxicity, as indicated by the reduction in optical density (OD 495) values, which reflect cell proliferation. As expected, [Bpy]Br demonstrated higher (p<0.05) cytotoxicity than [Bmim]Br, as evident from the sharper decline in OD 495 values at equivalent concentrations.

### Effects of [Bmim]Br and [Bpy]Br on cell motility

**Figure 2:**
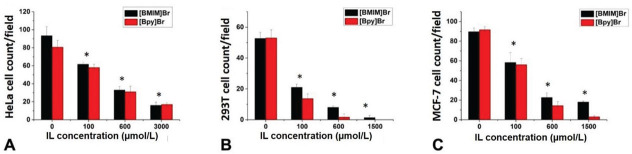
Motility of [Bmim]Br and [Bpy]Br in A) HeLa; B) HEK293T; and C) MCF-7 cells. * statistically significant difference from control (0 μmol/L) (p<0.05; Student’s *t*-test)

Figure 2 shows that the motility of HeLa, HEK293T, and MCF-7 cells was affected by [Bmim]Br and [Bpy]Br in a concentration-dependent manner. [Bmim]Br and [Bpy]Br showed comparable inhibitory trends, with slight variations depending on the cell type and the applied IL, possibly due to variations in their physiological responses to these compounds.

### Apoptotic effects of [Bmim]Br and [Bpy]Br

Bmim]Br and [Bpy]Br induced concentration-dependent apoptotic effects in HeLa and MCF-7 cell lines. Treatment with either IL significantly reduced the percentage of living cells and increased apoptotic (early and late) and dead cell populations as the concentration increased (Table 3).

**Table 3:** Percentage of apoptotic cells in HeLa and MCF-7 human cancer cell lines treated with [Bmim]Br and [Bpy]Br

**Treatment type**	**Cell type**	**Concentration (μmol/L)**	**Cell percentage (%)**			
			**Living cells**	**Early apoptotic**	**Late apoptotic**	**Dead cells**
[Bmim]Br	HeLa	0	95.50	1.45	2.45	0.60
		100	79.80*	5.50*	14.05*	0.65
		1500	47.53*	8.52*	42.15*	1.79*
	MCF-7	0	90.40	1.80	6.95	0.85
		100	61.28*	2.00	35.33*	1.40*
		1500	41.88*	5.00*	50.62*	2.50*
[Bpy]Br	HeLa	100	79.60*	4.93*	15.13*	0.35
		1000	38.32*	8.98*	50.90*	1.80*
	MCF-7	100	55.79*	4.47*	36.68*	1.05
		1000	33.86*	3.94*	60.63*	1.57*

* significant difference from control (0 μmol/L) (p<0.05; Student’s *t*-test)

In HeLa cells, [Bmim]Br treatment caused a significant increase (p<0.05) in late apoptotic cells, from 2.45 % at 0 μmol/L to 42.15 % at 1500 μmol/L. The number of early apoptotic cells also increased but to a lesser extent (from 1.45 % to 8.52 %). [Bpy]Br showed a similar trend, with late apoptotic cells increasing dramatically from 15.13 % at 100 μmol/L to 50.90 % at 1000 μmol/L, indicating a strong induction of apoptosis compared to [Bmim]Br.

In MCF-7 cells, [Bmim]Br also caused a significant increase (p<0.05) in late apoptotic cells from 6.95 % (0 μmol/L) to 50.62 % (1500 μmol/L), while [Bpy]Br exhibited an even greater apoptotic effect, with late apoptotic cells increasing from 36.68 % at 100 μmol/L to 60.63 % at 1000 μmol/L and a corresponding decrease in live cells from 55.79 % to 33.86 %.

### Effects of [Bmim]Br and [Bpy]Br on cyclin gene transcription

**Figure 3:**
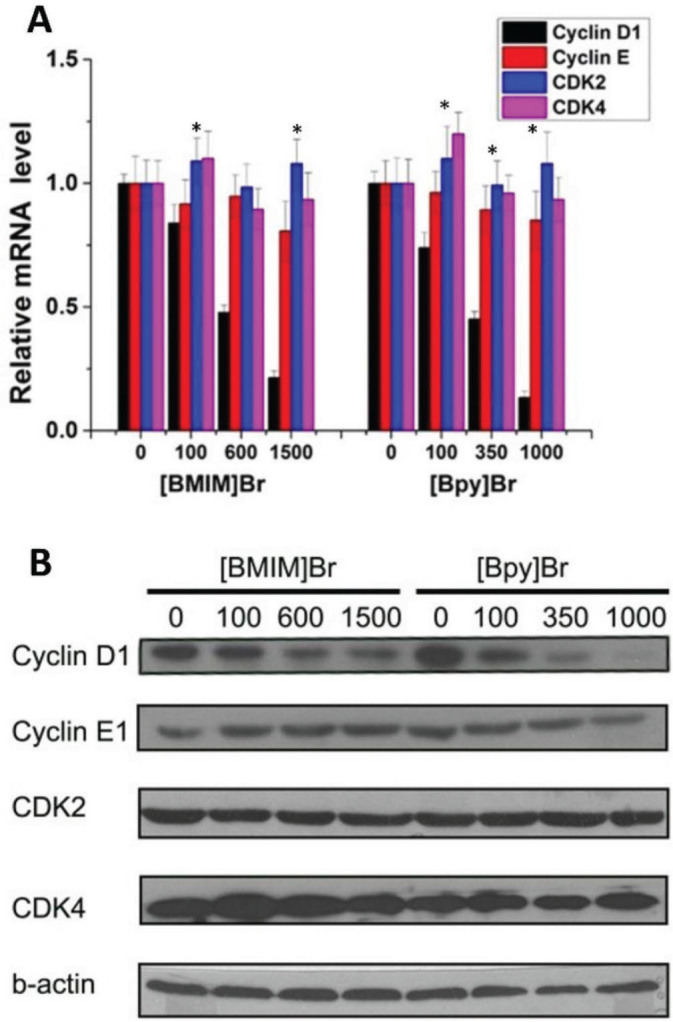
[Bmim]Br and [Bpy]Br effects on mRNA (panel A) and protein levels (panel B) of different cyclins and CDKs in HeLa cells. * statistically significant difference from control (0 μmol/L) (p<0.05; Student’s *t*-test)

The mRNA expression of cyclins D1 and E1 dropped significantly in cells treated with increasing concentrations of [Bmim]Br and [Bpy]Br than in respective controls, whereas the in changes kinases CDK2 and CDK4 were less pronounced (Figure 3). Cyclin D1 and E1 reductions were concentration-dependent. Our findings suggest that [Bmim]Br and [Bpy]Br inhibit cell cycle progression by suppressing these two key regulatory genes required for the G1-S phase transition. This inhibitory effect is more noticeable with [Bpy]Br than [Bmim]Br at equivalent concentrations, highlighting its stronger anti-proliferative potential.

**Figure 4:**
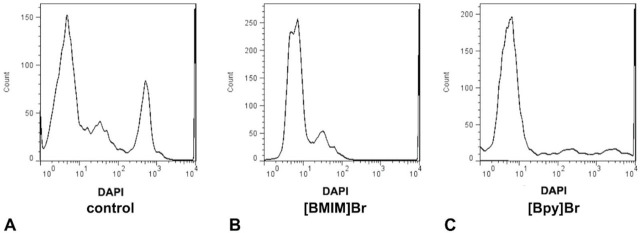
HeLa cell cycle upon treatment with [Bmim]Br and [Bpy]Br: A) normal cell-cycle distribution; B) [Bmim]Br-treated cells showing G0/G1 phase arrest; and C) [Bpy]Br-treated cells with enhanced G0/G1 phase arrest

Additionally, DAPI histograms show disruption of the cell cycle upon treatment with [Bmim]Br and [Bpy]Br compared to the control (Figure 4). Both ILs caused cell cycle arrest, as evidenced by the accumulation of cells in specific phases of the cell cycle, but the effect was more pronounced with [Bpy]Br, which is consistent with its stronger downregulation of cyclin gene expression.

## DISCUSSION

Our study has confirmed the cytotoxic effects of ILs already reported in various living cells (9, 29–30). Both [Bmim]Br and [Bpy] Br exhibited acute and long-term cytotoxicity in a concentration-dependent manner, but the latter was more toxic. Although our ILs do not vary in alkyl chain length, this difference in effect may be owed to differences in cationic structures that interact differently with cell membranes. Pérez et al. (29) reported that variations in ionic structures, such as those between monocationic and dicationic, can influence toxicity across different cell types. However, both [Bmim]Br and [Bpy]Br in our study are monocationic, and the observed differences in cytotoxicity may primarily be owed to different interactions between the imidazolium and pyridinium head groups with cellular components, membranes in particular.

In terms of cell proliferation, HeLa, HEK293T, and MCF-7 cell lines showed varying sensitivity to [Bmim]Br and [Bpy]Br over four days, which could stem from differences in cell membrane composition or oxidative stress defence mechanisms. These variations echo the tissue-specific responses reported in liver cancer cells (31) and *Daphnia magna* (32).

While various compounds can induce apoptosis via common pathways, including pro-apoptotic factor regulation, our findings point to a distinct mechanism of action affecting cell motility, a critical biological process underlying wound healing, immune response, and cancer metastasis, reported earlier (33, 34). In our study, both monocationic ILs impeded cell motility in both cancer cell types and HEK293T in a concentration-dependent manner, [Bpy]Br more than [Bmim]Br, which aligns with reports of similar studies of pyridinium- and imidazolium-based ILs in HeLa and MCF-7 cell lines. Namely, Pérez et al. (29) reported notably higher cytotoxicity of monocationic than dicationic pyridinium-based ILs (like our [Bpy]Br) – attributed to their greater lipophilicity and membrane-disruptive potential – whereas Kuczak et al. (25) reported a strong antitumor activity of imidazolium-triflate ILs (like our [Bmim]Br) in MCF-7 cells – exceeding even that of cisplatin – which they linked to their ability to disrupt redox balance, trigger apoptosis, and cause cell cycle arrest.

As for the generally stronger effects of [Bpy]Br than [Bmim]Br in our study, they may be owed to the difference in their cationic parts. Toxicological studies across various cell lines have demonstrated that cationic moieties, particularly the structure of the head group, play a crucial role in determining cytotoxicity (35). The cationic head groups of [Bpy]Br (butylpyridinium) and [Bmim] Br (butylmethylimidazolium) have similar cytotoxic profiles but vary in ring structure and charge distribution. The planar pyridinium ring of [Bpy]Br tends to interact more strongly with cellular membranes than the imidazolium ring of [Bmim]Br (29). However, different sensitivity observed between HeLa and MCF-7 cells suggests that cell-specific responses may be influenced by intrinsic factors such as membrane composition, apoptotic signalling pathways, or metabolic characteristics unique to each cell line. Such variability calls for tailoring anti-cancer therapeutic approaches to the cellular context. According to Beaven et al. (34), ILs are capable of improving the delivery of both small and large molecules due to their unique physicochemical properties and ease of functionalisation, which can help overcome biological and physical barriers. ILs can enhance the therapeutic efficacy of anti-tumour agents and offer novel approaches to combined treatment of chronic diseases like cancer (36).

An exemplary novel approach is the synthesised [Py-2OH]OAc obtained by sonicating pyridine with 2-chloropropane-1,3-diol by Nayl et al. (37), which proved to be advantageous over traditional strategies and a potent anticancer agent against MCF-7 cells. The apoptotic effect was dose-dependent, similar to the ILs in our study, with higher concentrations leading to significant cell death and apoptosis in MCF-7 cells. In their study of pyridinium-based ILs, Pérez et al. (29) reported significantly reduced mitochondrial membrane potential as a trigger to apoptotic cascade in various cancer cell types. This mechanism of action may have also taken place with [Bmim]Br and [Bpy]Br, and looking into it further could provide a better insight into their potential as therapeutic agents or cytotoxic compounds (38).

In terms of cell-cycle disruption, which is a desired effect to stop cancer cell proliferation, our two ILs were effective arresting the cell cycle in the G0/G1 phase, which was accompanied by a substantial drop in cyclin D1 expression. These findings align with earlier reports of arrested cell cycle by ILs (39) and of the direct link between cyclin expression and impaired cell cycle progression (40). Kyca et al. (41), in turn, highlighted the capacity of ILs to induce oxidative stress as a mechanism of disrupting cell cycle, but we did not look into oxidative stress parameters.

## CONCLUSIONS

This study demonstrates that [Bmim]Br and [Bpy]Br exert concentration-dependent cytotoxicity, induce apoptosis, and disrupt cell cycle progression in HeLa and MCF-7 cancer cell lines. Furthermore, stronger [Bpy]Br cytotoxicity may be attributed to structural variations in their cationic head groups, influencing their interactions with cellular membranes. While these findings provide important insights into the toxicological profiles of ionic liquids, additional studies comparing their effects on non-cancerous human cells are needed to fully assess their therapeutic potential and biomedical application.
